# Non-task expert physicians benefit from correct explainable AI advice when reviewing X-rays

**DOI:** 10.1038/s41598-023-28633-w

**Published:** 2023-01-25

**Authors:** Susanne Gaube, Harini Suresh, Martina Raue, Eva Lermer, Timo K. Koch, Matthias F. C. Hudecek, Alun D. Ackery, Samir C. Grover, Joseph F. Coughlin, Dieter Frey, Felipe C. Kitamura, Marzyeh Ghassemi, Errol Colak

**Affiliations:** 1 LMU Center for Leadership and People Management, Department of Psychology, LMU Munich, Munich, Germany; 2 Department of Infection Prevention and Infectious Diseases, University Hospital Regensburg, Regensburg, Germany; 3 MIT Computer Science & Artificial Intelligence Lab, Massachusetts Institute of Technology, Cambridge, MA USA; 4 MIT AgeLab, Massachusetts Institute of Technology, Cambridge, MA USA; 5 Department of Business Psychology, University of Applied Sciences Augsburg, Augsburg, Germany; 6 Department of Psychology, LMU Munich, Munich, Germany; 7 Department of Experimental Psychology, University of Regensburg, Regensburg, Germany; 8 Department of Emergency Medicine, St. Michael’s Hospital, Unity Health Toronto, Toronto, Canada; 9 Division of Emergency Medicine, University of Toronto, Toronto, Canada; 10 Li Ka Shing Knowledge Institute, St. Michael’s Hospital, Unity Health Toronto, Toronto, Canada; 11 Division of Gastroenterology, St. Michael’s Hospital, Toronto, Canada; 12 Departamento de Diagnóstico por Imagem, Universidade Federal de São Paulo, São Paulo, Brazil; 13 DasaInova, Dasa, São Paulo, Brazil; 14 Electrical Engineering and Computer Science, Institute for Medical Engineering and Science, Massachusetts Institute of Technology, Cambridge, MA USA; 15 Vector Institute, Toronto, Canada; 16 Department of Medical Imaging, St. Michael’s Hospital, Unity Health Toronto, Toronto, Canada; 17 Department of Medical Imaging, Faculty of Medicine, University of Toronto, Toronto, Canada

**Keywords:** Human behaviour, Medical imaging, Computer science

## Abstract

Artificial intelligence (AI)-generated clinical advice is becoming more prevalent in healthcare. However, the impact of AI-generated advice on physicians’ decision-making is underexplored. In this study, physicians received X-rays with correct diagnostic advice and were asked to make a diagnosis, rate the advice’s quality, and judge their own confidence. We manipulated whether the advice came with or without a visual annotation on the X-rays, and whether it was labeled as coming from an AI or a human radiologist. Overall, receiving annotated advice from an AI resulted in the highest diagnostic accuracy. Physicians rated the quality of AI advice higher than human advice. We did not find a strong effect of either manipulation on participants’ confidence. The magnitude of the effects varied between task experts and non-task experts, with the latter benefiting considerably from correct explainable AI advice. These findings raise important considerations for the deployment of diagnostic advice in healthcare.

## Introduction

The number of artificial intelligence (AI) enabled software applications for radiology is growing rapidly. By now, there are more than 190 CE-marked products available, from which almost 100 have received Class II or Class III FDA clearance (https://www.AIforRadiology.com). For chest X-rays, which are the most frequently performed radiological examination worldwide^1^, there is a wide array of certified AI-enabled clinical decision support systems (AI-CDSS) on the market^2^. Many AI models developed for radiology tasks have shown excellent performance equal to or even surpassing human experts (e.g.^3,4^), but few studies have investigated these products' actual clinical impact (e.g., physicians’ diagnostic performance and patient outcomes) when implemented in a natural clinical setting^2^. The limited work that does investigate whether AI-CDSS have actual clinical benefits is inconclusive^2,5,6^, suggesting that the technology might not automatically lead to better patient outcomes.

One reason for the rather limited effectiveness of AI-CDSS in deployment may be a difference in usage: while these systems are evaluated based on their predictions in isolation, in practice they are most often used in conjunction with a human intermediary. As long as AI-enabled radiology software does not autonomously diagnose or classify findings, the predictions from the models have to be regarded as diagnostic advice which can be accepted or rejected by a physician who has to make the final decision. However, at the moment, research on how users interpret and act on AI-generated advice is limited. Previous studies have shown that people often rely heavily on any given advice and even fail to dismiss inaccurate advice. This has been shown both in clinical tasks among physicians^7,8^ and in other decision-making scenarios^9^. Knowing that physicians can be influenced by advice, it is crucial to study the optimal way of presenting clinical advice to maximize its efficacy.

To this end, one promising direction for helping users contextualize and better incorporate AI-generated advice is making the inner workings and decision criteria of AI models more transparent^10^. Providing additional reasoning for AI recommendations (e.g., visual annotations on X-rays) could potentially help mitigate over-reliance and encourage appropriate trust^11^. Previous research has shown that providing case-by-case explanations indeed increases trust in and reliance on the advice, even when the advice is incorrect^8^. However, it is less well understood how the explainability of advice affects users with different levels of task expertise. For instance, it is plausible that physicians who receive less specialized training than radiologists to review medical images might benefit more from diagnostic advice alongside a visible annotation on the image indicating what region influenced the advice. To the best of our knowledge, the question of whether explanations affect the diagnostic decisions of physicians with different amounts of task expertise has not yet been studied.

Several studies have compared diagnostic performance when reviewing images with and without AI support (see^12^ for a systematic review in CT and chest X-rays). However, in a clinical setting, physicians often receive advice from colleagues, or are asked to re-review cases from someone else to give a second opinion^13,14^. Therefore, comparing different forms of advice—e.g., AI-generated or from a colleague—is more pertinent to fully understand the unique impact of AI-enabled CDSS on clinical decision-making. Indeed, verifying and falsifying suggestions is a different cognitive task than independently gathering findings and deducing a diagnosis^15,16^. Therefore, comparing diagnostic performance when reviewing cases with and without AI support, while extremely interesting, tests different underlying decision-making processes. When comparing different forms of advice, studies have found both *algorithmic aversion* (i.e., preferring human advice compared to an algorithm, (e.g.^17^)) and *algorithmic appreciation* (i.e., preferring advice from an algorithm compared to human advice (e.g.^18^)). These varying observations might be due to several factors—for instance, it has been shown that people with high task expertise are more inclined to dismiss or devalue task-related advice from an AI system than are people with low task expertise^7,18^. It has also been shown that even when participants rate the quality of AI advice as lower than human advice, they still follow both sources of advice to the same degree^7,9^. Given this discrepancy between the evaluation of and the reliance on the advice, testing the influence of the source of advice on physicians’ confidence in their final diagnostic decision is also pertinent.

**Figure 1:**
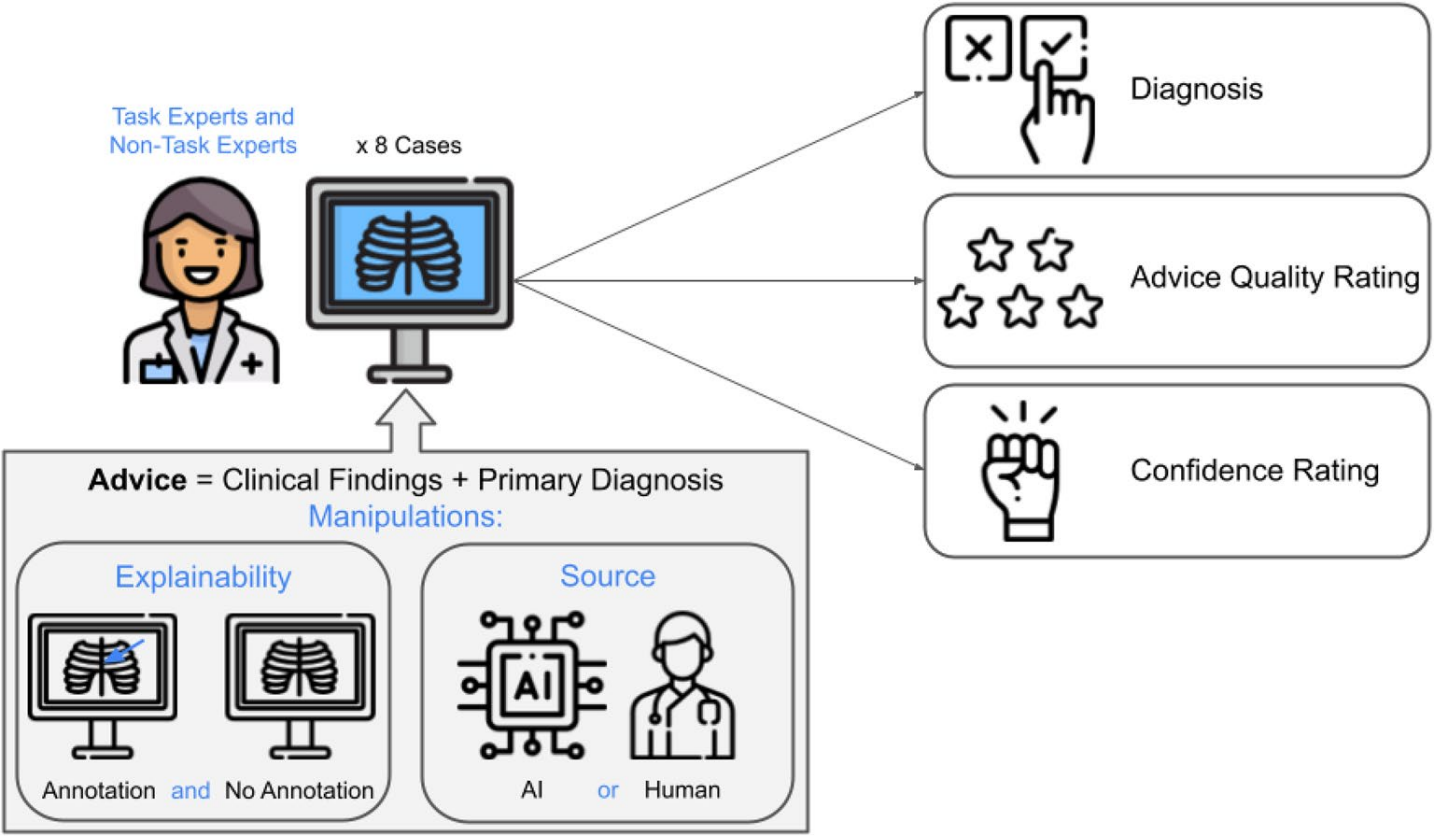
Experimental setup. Every participant reviewed all eight cases. Each case consisted of a brief patient vignette, a chest X-ray, and diagnostic advice (radiologic findings and primary diagnoses). The advice came either with or without annotations on the X-ray. Additionally, the advice was labeled as coming either from an AI system or an experienced radiologist. Physicians were asked to give a final diagnosis, rate the quality of the advice, and judge how confident they were with their diagnosis.

In the present study, physicians with different levels of task expertise (i.e., task experts and non-task experts) were asked to review a series of chest X-rays since this is a prevalent diagnostic task for which AI technology is widely applicable. The participants received only accurate diagnostic advice (verified by experts), with two levels of explainability by either showing visual annotations indicating where the proposed condition can be seen on the X-rays or presenting the X-rays without an annotation. Additionally, the purported source of advice was labeled as coming either from an AI system or a human radiologist. We tested the impact of these two advice manipulations (explainability and source) on three dependent variables: physicians’ (1) diagnostic accuracy, (2) ratings of advice quality, and (3) confidence in their final diagnosis. Figure 1 gives an overview of the experimental design.

## Results

### Analysis

We calculated three mixed-effects regression models, one for each dependent variable: (1) diagnostic accuracy, (2) advice quality ratings, and (3) confidence in the diagnosis. The equations corresponding to the statistical model outputs can be found in the online supplements (https://osf.io/h7aj3/). The diagnostic accuracy was assessed using a logistic regression model because it was measured as a binary variable (accurate/inaccurate). Linear regression models were applied for the advice quality and the confidence ratings. Each dependent variable was regressed on the explainability of the advice (annotated vs. non-annotated), the source of the advice (AI vs. human), the task expertise (radiologists vs. IM/EM physicians), the interaction between explainability (annotated vs. non-annotated) and source (AI vs. human), and the control variables (professional identification, belief in professional autonomy, self-reported AI knowledge, attitude toward AI technology, and years of professional experience). All models included fixed effects for all variables mentioned above and a random effect for the participants to account for non-independence of observations and differences in their skills, as well as a random effect for the patient cases to account for their different difficulty levels. Further, we chose mixed-effects regression models because they are particularly useful for analyzing experiments with a repeated measures design. One of the eight cases, which had been taken from the previous study without changes, had no clinical abnormalities (diagnosis: normal) and, consequently, no annotations on the image. A second case was shown without annotations due to a technical issue. These two cases had to be excluded from the analysis because the explainability condition could not be unambiguously assigned.

### Non-task experts benefited from correct explainable AI advice

**Figure 2:**
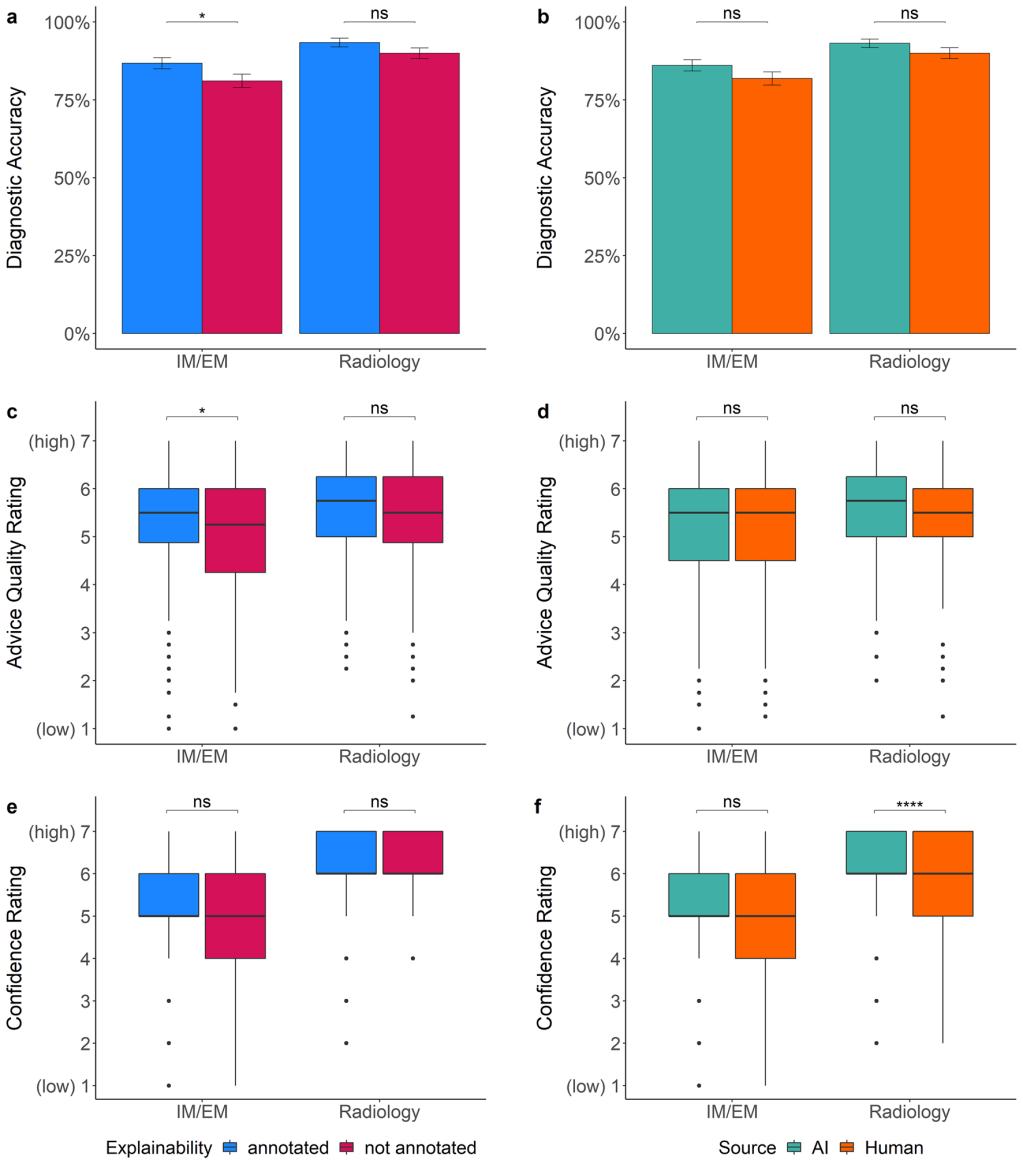
Dependent variables across the advice manipulations. The plots show how the advice manipulations affected non-task experts (i.e., IM/EM physicians) and task experts (i.e., radiologists). Plot (**a**) shows that explainable advice helped non-task experts to be more accurate on average (*p*_*IM/EM*_ = 0.042, *p*_*Radiology*_ = 0.120). Chart (**b**) indicates that the source of advice had only statistically non-significant effects on diagnostic accuracy (*p*_*IM/EM*_ = 0.129, *p*_*Radiology*_ = 0.155). Plot (**c**) displays that non-task experts rated the quality of annotated advice higher than non-annotated advice (*p*_*IM/EM*_ = 0.011, *p*_*Radiology*_ = 0.195). In (**d**), it is shown that there was no evidence that the source of advice had an effect on the quality rating (*p*_*IM/EM*_ = 0.645, *p*_*Radiology*_ = 0.812). Plot (**e**) indicates that explainability had little effect on the confidence ratings (*p*_*IM/EM*_ = 0.280, *p*_*Radiology*_ = 0.202). Finally, (**f**) shows that task experts reported higher confidence in their decision when receiving AI advice while non-task experts’ confidence was unaffected by the source (*p*_*IM/EM*_ = 0.497, *p*_*Radiology*_ < 0.0001). The boxplots show 25th to 75th percentiles and the median as the central line; the whiskers extend to a maximum of 1.5 × interquartile range. The error bars represent standard errors. *p ≤ 0.05, **p ≤ 0.01, ***p ≤ 0.001, ****p ≤ 0.0001, *ns* statistically non-significant.

First, we tested whether the participants’ diagnostic accuracy was influenced by the experimental manipulations (see Table 1). Task experts (i.e., radiologists) performed significantly better than non-task experts (i.e., IM/EM physicians). Overall, participants showed a higher diagnostic accuracy when they received advice with an explanation (i.e., an annotation). They also performed better when the advice was labeled as coming from the AI instead of the human. When looking at the results separated by task expertise (defined by discipline), we found that providing annotated advice improved the mean performance of the IM/EM physicians in our experiment by 5.66% (*p* = 0.042, see Fig. 2a). The diagnostic performance of the radiologists was 3.41% better when receiving annotated advice. However, the mean difference was statistically non-significant (*p* = 0.120, see Fig. 2a). Non-experts performed 4.22% better and experts 3.15% better when receiving advice labeled as coming from the AI (see Fig. 2b), but both mean differences were statistically non-significant (*p*_*IM/EM*_ = 0.129, *p*_*Radiology*_ = 0.155). Moreover, higher self-reported AI knowledge was generally associated with better task performance (see Table 1). It should be noted that task experts rated their self-reported AI knowledge significantly higher than non-task experts (Table S2), which might explain this result.

**Table 1:** Logistic mixed multilevel regression models for participants’ diagnostic accuracy.

Predictors	Odds ratios	SE	95% CI	z	p
Intercept	5.86	2.89	2.23–15.40	3.59	** < 0.001**
Explainability (annotated)	2.30	0.60	1.37–3.85	3.16	**0.002**
Source (AI)	2.10	0.58	1.22–3.59	2.69	**0.007**
Task expertise (experts: radiologists)	2.20	0.51	1.40–3.47	3.41	**0.001**
Professional identification	1.00	0.12	0.80–1.26	0.03	0.980
Beliefs about professional autonomy	1.20	0.14	0.95–1.52	1.57	0.117
Self-reported AI-knowledge	1.34	0.19	1.01–1.77	2.03	**0.042**
Attitude toward AI	0.99	0.12	0.78–1.26	−0.06	0.953
Professional experience (years)	0.99	0.01	0.97–1.01	−1.11	0.267
Explainability (annotated) × source (AI)	0.57	0.22	0.27–1.20	−1.48	0.139

Random effects: σ^2^ = 3.29, τ_00 ID_ = 0.60, τ_00 PATIENTID_ = 1.10, ICC = 0.34, *N*_ID_ = 222, *N*_PATIENTID_ = 6, observations = 1332, marginal R^2^ = 0.086/conditional R^2^ = 0.397; OR > 1 variable associated with higher odds for correct diagnosis; OR < 1 variable associated with lower odds for correct diagnosis, OR = 1 variable does not affect odds of outcome. The intercept indicates that the probability of an accurate diagnosis was 0.85 when all predictors are zero. Predictors without a natural zero point (i.e., professional identification, beliefs about professional autonomy, self-reported AI-knowledge, attitude toward AI) were mean-centered.

*SE* standard error, *p* probability of committing a type I error.

Statistically significant values are in bold.

### Non-task experts preferred explainable advice

Next, we examined whether the experimental manipulations influenced participants’ advice quality rating (see Table 2). Participants rated the quality of the advice higher, on average, if it was given with an explanation, i.e., with a visible annotation on the X-ray. When comparing the results by task expertise, we saw that annotated advice led to a 0.24 point (on a scale from 1 to 7) higher mean advice quality rating among non-task experts (*p* = 0.011, see Fig. 2c). Task experts rated the quality of the annotated advice on average 0.11 points higher, but the mean difference was statistically non-significant (*p* = 0.195, see Fig. 2d). The effect of the source of the advice on the advice quality rating was statistically non-significant for both groups (*p*_*IM/EM*_ = 0.645, *p*_*Radiology*_ = 0.812). Non-task experts rated the quality advice labeled as coming from the AI 0.04 points lower and task-experts 0.02 points higher. Overall, participants with higher task expertise rated the quality of the advice significantly higher than non-task experts. Attitude toward AI technology was the only other significant (positive) predictor for the quality rating in the overall sample (see Table 2).

**Table 2:** Linear mixed multilevel regression models for advice quality rating.

Predictors	Estimate	SE	95% CI	t	p
Intercept	5.17	0.20	4.78 to 5.55	26.23	** < 0.001**
Explainability (annotated)	0.25	0.07	0.11 to 0.39	3.42	**0.001**
Source (AI)	0.03	0.11	−0.19 to 0.25	0.29	0.771
Task expertise (experts: radiologists)	0.24	0.10	0.04 to 0.45	2.32	**0.020**
Professional identification	0.03	0.05	−0.07 to 0.14	0.59	0.554
Beliefs about professional autonomy	−0.09	0.05	−0.20 to 0.01	−1.70	0.089
Self-reported AI-knowledge	0.07	0.07	−0.06 to 0.20	1.08	0.279
Attitude toward AI	0.13	0.06	0.02 to 0.23	2.27	**0.023**
Professional experience (years)	−0.00	0.01	−0.02 to 0.01	−0.90	0.367
Explainability (annotated) × source (AI)	−0.07	0.10	−0.27 to 0.12	−0.75	0.454

Random effects: σ^2^ = 0.79, τ_00 ID_ = 0.42, τ_00 PATIENTID_ = 0.16, ICC = 0.42, *N*_ID_ = 222, *N*_PATIENTID_ = 6, observations = 1332, marginal R^2^ = 0.042/conditional R^2^ = 0.449. The regression estimate indicates how much the mean quality rating changes given a one-unit shift in the predictor while holding other predictors in the model constant. The intercept represents the mean value of the advice quality rating when all predictor variables are zero. Predictors without a natural zero point (i.e., professional identification, beliefs about professional autonomy, self-reported AI-knowledge, attitude toward AI) were mean-centered.

*SE* standard error, *p* probability of committing a type I error.

Statistically significant values are in bold.

### AI advice boosted task experts’ confidence in their diagnosis

When looking at participants’ confidence in their diagnostic decisions, task experts (i.e., radiologists), as expected, reported being more confident with their diagnosis (see Table 3). We could only find statistically non-significant associations between explainability, as well as the source of the advice, and participants’ confidence rating in the combined sample. When comparing task experts with non-task experts, receiving X-rays with annotations had little influence on participants’ mean confidence (± 0.10 points on a 7-point Likert scale) in either group (*p*_*IM/EM*_ = 0.280, *p*_*Radiology*_ = 0.202, see Fig. 2e). However, radiologists reported 0.32 points higher mean confidence in their final diagnosis when they received advice labeled as coming from the AI vs. the human (*p* < 0.0001, see Fig. 2f). IM/EM physicians rated their mean confidence only 0.06 points higher when receiving advice supposedly coming from the AI (*p* = 0.497). The only other variable that was statistically significantly associated with being more confident in their diagnosis was higher self-reported AI knowledge (see Table 3). As mentioned above, the fact that task experts rated their self-reported AI knowledge higher than non-task experts (Table S2), might explain this result.

**Table 3:** Linear mixed multilevel regression models for confidence in the diagnosis.

Predictors	Estimate	SE	95% CI	t	p
Intercept	5.10	0.19	4.73 to 5.48	26.77	** < 0.001**
Explainability (annotated)	0.06	0.07	−0.09 to 0.20	0.77	0.440
Source (AI)	0.19	0.10	−0.01 to 0.39	1.91	0.056
Task expertise (experts: radiologists)	0.72	0.09	0.54 to 0.89	7.93	** < 0.001**
Professional identification	−0.00	0.05	−0.09 to 0.09	−0.05	0.956
Beliefs about professional autonomy	0.01	0.05	−0.09 to 0.10	0.16	0.874
Self-reported AI-knowledge	0.19	0.06	0.08 to 0.30	3.39	**0.001**
Attitude toward AI	−0.01	0.05	−0.11 to 0.08	−0.26	0.794
Professional experience (years)	0.01	0.00	−0.00 to 0.02	1.79	0.074
Explainability (annotated) × source (AI)	−0.03	0.10	−0.23 to 0.17	−0.32	0.749

Random effects: σ^2^ = 0.85, τ_00 ID_ = 0.27, τ_00 PATIENTID_ = 0.16, ICC = 0.34, N _ID_ = 222, N _PATIENTID_ = 6, observations = 1332, marginal R^2^ = 0.130/conditional R^2^ = 0.424. The regression estimate indicates how much the mean confidence rating changes given a one-unit shift in the predictor while holding other predictors in the model constant. The intercept represents the mean value of the confidence in the diagnosis when all predictor variables are zero. Predictors without a natural zero point (i.e., professional identification, beliefs about professional autonomy, self-reported AI-knowledge, attitude toward AI) were mean-centered.

*SE* standard error, *p* probability of committing a type I error.

Statistically significant values are in bold.

### Performance across clinical cases

**Figure 3:**
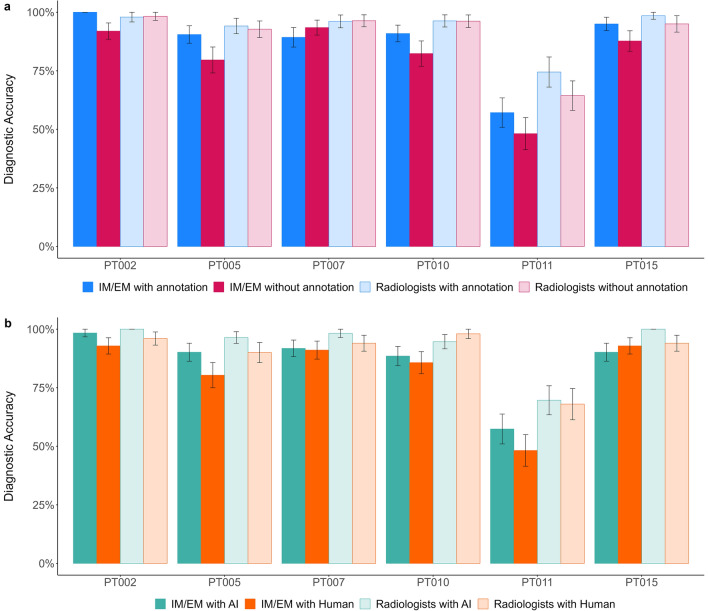
Diagnostic accuracy by clinical case. Case-dependent performance amongst non-task experts (i.e., IM/EM physicians) and task experts (i.e., radiologists) across the two advice manipulations (**a**) explainability of the advice and (**b**) source of the advice. The x-axis labels are the case ID numbers (see online supplements pages 2–4 for further information about the cases). The error bars represent standard errors.

Finally, we also looked at participants’ task performance for each clinical case (see Fig. 3). Overall performance was high. However, it was much lower for Case ID PT011 under both advice manipulations. This finding is consistent with our previous study using the same patient cases^7^. While annotations on the X-rays had only a limited benefit for the task experts across all cases, for this more difficult case, annotations seem to have had a substantially greater positive effect even on task experts. Among IM/EM physicians, receiving annotated advice was generally associated with higher diagnostic accuracy (except for case PT007, which showed the smallest difference between the two conditions). Interestingly, non-task experts’ performance was on par with experts in the annotated condition. This might indicate that non-task experts benefit more from explainable advice independent of case complexity. Across all cases, the source of the advice had a rather little effect on radiologists’ performance. Non-task experts showed slightly better performance (to a varying degree) when receiving advice labeled as coming from an AI across all but one case (PT015). As depicted in Fig. 3, two cases (PT002 and PT015) had only limited performance variations under certain conditions. Therefore, we reran all three regressions without these cases and confirmed that the results were stable. The results of these additional regressions can be found in the online supplements (https://osf.io/h7aj3/).

## Discussion

AI-enabled clinical decision support systems (AI-CDSS) are increasingly being implemented in healthcare facilities to improve efficiency and patient outcomes^2,6^. For the foreseeable future, predictions by an AI-CDSS will be seen as advice to support physicians in making diagnostic decisions. However, the underlying mechanisms of how the presentation of diagnostic advice affects physicians’ decision-making are still understudied. Considering the potential adverse consequences of implementing suboptimally designed AI-CDSS, the present study aimed to examine some of these mechanisms systematically. The findings raise important considerations for the use of AI-generated advice.

The results indicate that having an explanation (i.e., annotation on the X-ray indicating the area that determined the prediction) as part of the advice positively affected physicians' diagnostic accuracy and their quality rating. Several findings stand out when looking at the impact of explainability of advice. First, when comparing task experts (i.e., radiologists) with non-task experts (i.e., IM/EM physicians), the latter group significantly benefited from the annotations while the effect was statistically non-significant for the task experts. There are at least two probable explanations for this. On one hand, since the overall performance of the radiologists was very high, it is possible that task experts also profited from seeing the annotations, but ceiling effects masked these benefits. This assumption is supported by looking at the individual patient cases, where we see that task experts indeed profited more from visual annotations when reviewing more challenging cases. Therefore, future studies could include even more complex cases to examine this further. However, because trained radiologists are expected to perform well on a radiology task, making material artificially more challenging might reduce external validity. On the other hand, it is also possible that task experts do not need visual explanations on the X-rays when already receiving findings and a primary diagnosis since this information is sufficient to guide their attention to the critical area on the image. Similarly, it is also feasible that written findings and diagnoses could be less easily comprehensible than a simple visual annotation for non-task experts. It would be interesting to record the reading time per case as a secondary outcome variable to examine the effects of explainable advice further. Reading time might decrease significantly by having annotations both for task-experts and non-experts. Moreover, it is surprising that annotations had little effect on participants’ confidence in their own final diagnosis. We assumed that highlighting the area which determined the advice’s prediction would make participants more confident in accepting or rejecting the proposed diagnosis^19^. Considering the high confidence level, on average, among task experts, ceiling effects might again explain this surprising result among the radiologists. However, non-task experts, who did not report particularly high levels of confidence on average, also gained very little from receiving visual annotations on the X-rays. This is especially surprising since their diagnostic accuracy and advice quality rating were higher in this condition. More research is needed to better understand which factors influence decision confidence among physicians when receiving diagnostic advice.

The findings from the present study indicate that the source of the advice being labeled as coming either from an AI system or a human radiologist influenced participants’ decision making. Overall, physicians’ diagnostic accuracy was higher when they received AI advice. Surprisingly, the advice quality rating was only marginally affected by both sources of advice, which means that we neither found convincing evidence for algorithmic appreciation nor for algorithmic aversion. This finding does not confirm the results from a previous study using the same materials, in which task experts did show algorithm aversion by rating the quality of AI advice lower than human advice^7^. We hypothesized that this slight inconsistency could be caused by differences in attitude toward AI technology or self-reported AI knowledge among the two samples; however, this was not backed by the data (see Table S3 and Table S4 for a sample comparison). In the previous study, participants were shown both correct and incorrect advice, which is another potential reason for variation in the results. Research has shown that people are less willing to forgive an error made by an AI compared to a human^17^. And indeed, when comparing the data from the cases with correct advice without annotations (the only comparable group between the two studies), we found that participants in the previous study rated the quality of AI advice (*M* = 4.96, *SD* = 1.21) significantly lower than did participants in the present study (*M* = 5.28, *SD* = 1.17). At the same time, the quality rating for the human advice in the previous (*M* = 5.28, *SD* = 1.29) and the present study (*M* = 5.26, *SD* = 1.32) was almost the same. This finding confirms results from other research indicating that people are less lenient with an AI when it errs. We were also surprised to find that receiving advice from the AI compared to human advice was followed by higher confidence ratings among task experts. Their higher confidence in the AI condition was not reflected by statistically significant differences in diagnostic accuracy. However, as with the explainability manipulation, ceiling effects might have masked trends. Task experts might assume that the AI system uses a different reviewing approach to a human and, therefore, could have access to additional information unknown to them. Consequently, when the AI comes to the same conclusion as they do, it might be a more valuable confirmation and validation of their decision for the task experts. At the same time, non-task experts might perceive the advice from both an AI and a human expert to be of equal value.

Finally, we want to highlight that receiving annotated advice from an AI led to the highest diagnostic accuracy among non-task experts (*M* = 86.84, *SD* = 33.89) and task experts (*M* = 94.61, *SD* = 22.64). It is noteworthy that non-task experts’ performance in the annotated AI condition was almost on par with task experts’ performance in the not-annotated human condition (*M* = 88.00, *SD* = 32.60). These findings underline the idea that non-task experts such as IM and EM physicians may especially benefit from implementing AI-CDSS for image reviewing tasks. In hospitals, physicians not trained in radiology often have to read X-rays and diagnose patients without radiology reporting during night or weekend shifts. Moreover, in many rural areas, even in highly developed countries, outside of regular business hours, expert radiology reporting is not available at all. Consequently, utilizing AI-CDSS, especially for IM and EM physicians, may help to increase patient flow efficiency and to provide good and safe patient care. However, this assumption will only result in better clinical outcomes when the AI-CDSS is highly accurate in its predictions. Our participants strongly relied on any advice given to them. We are confident in making this claim because participants often failed to dismiss inaccurate advice in the previous study, leading to lower overall performance^7^. When comparing the diagnostic accuracy between the two studies with the same condition (correct advice without annotation), we find a statistically non-significant difference (*t* = −1.71, *p* = 0.09). This result leads to the assumption that participants would have also failed to dismiss inaccurate advice in the present study. We decided to provide only correct advice in the present study because we wanted to test an ideal scenario where diagnostic advice should almost never fail. Considering that only eight cases were presented, presenting incorrect advice might have led participants to believe that the advisor was incompetent and dismiss the advice more often. Unfortunately, in reality, no AI-CDSS and no human radiologists have an accuracy rate of 100%. Therefore, more research is needed to test how advice with case-by-case explanations will affect physicians’ reliance on the advice when it is incorrect.

The present study has some limitations. First, the experiment was conducted online with participants knowing that their diagnosis would not affect the treatment decision for a real patient. The limited ability to capture decision risks in an experimental setup may have influenced physicians' reliance on the advice differently than would have been the case in a natural clinical setting. Second, participants only reviewed eight patient cases (and only six were included in the analysis), which somewhat narrows the generalizability of the results. However, it was necessary to keep the number of cases low to limit the duration of the experiment so that we could recruit the necessary number of physicians willing to complete the experiment. To overcome this limitation, our cross-institutional panel of radiologists selected cases to be representative of different levels of difficulty and clinical abnormalities. Third, we could not reach the sample size estimated through an a priori power analysis. While we planned to collect data from 128 IM/EM physicians and 128 radiologists, we ultimately could only recruit 117 and 106 participants in each group, respectively. Consequently, it is possible that we were not able to detect small effects (especially when comparing the two professional groups). Fourth, as already mentioned, diagnostic accuracy was high among the task experts across most cases, which limited the scope for improvement when receiving advice. We had expected that the radiologists would perform very well in the radiology task since we chose cases that would reflect the real clinical world for the study's external validity. However, even a small improvement in diagnostic accuracy in this setting would yield real-life clinical benefits and could potentially save lives. Finally, in the study invitations sent to institutions across the US and Canada, we asked people who had already participated in the previous study not to participate in the present study. Since the data is anonymized, we cannot rule out that a person might have participated in both studies. However, since the data for the present study were collected two years later, it is improbable that participants could recall the cases.

In conclusion, the fact that physicians benefitted the most from receiving explainable advice from an AI system underlines the potential opportunities that AI-enabled decision aids could have for the field of radiology and beyond. Our findings indicate that the implementation of AI-CDSS might be most valuable for non-radiologists when reviewing medical images and making timely clinical decisions without radiology reporting. This specific use case for AI-enabled clinical advice has a great potential for improving workflows, clinical outcomes, and patient safety. Further research should focus on how explainable AI advice has to be presented to non-task experts to optimize utility while minimizing blind over-reliance in the event that the AI-CDSS errs.

## Methods

### Participants

In total, *N* = 223 participants finished the online experiment and were included in the data analysis. The sample consisted of physicians with different levels of task expertise. On the one hand, physicians trained in internal medicine or emergency medicine (IM/EM) often review chest X-rays, but have relatively little formal training in viewing medical images and were consequently classified as non-task experts. Radiologists with specialized training in reviewing medical images were classified as task experts. Participants were recruited via email. Study invitations were sent to staff and residents at hospitals in the US and Canada and to residency program coordinators with the request to distribute the link. Table 4 displays the sample demographics. The study was exempt from a full ethical review by COUHES, the Institutional Review Board (IRB) for the Massachusetts Institute of Technology (MIT) because the research activities met the criteria for exemption as defined by Federal regulation 45 CFR 46. The experiment complied with all relevant ethical regulations and standards required by COUHES and the Ethical Principles of Psychologists and Code of Conduct outlined by the American Psychology Association (APA). Informed consent was obtained from all participants.

**Table 4:** Participant demographics.

	IM/EM (*N* = 117)	Radiology (*N* = 106)	Overall (*N* = 223)
Gender			
Female	46 (39.3%)	38 (35.8%)	84 (37.7%)
Male	66 (56.4%)	67 (63.2%)	133 (59.6%)
Other	1 (0.9%)	0 (0%)	1 (0.4%)
Prefer not to answer	4 (3.4%)	1 (0.9%)	5 (2.2%)
Professional experience (years)			
Mean (SD)	10.8 (9.95)	9.50 (8.96)	10.2 (9.49)
Median [min, max]	7.00 [0.500, 40.0]	6.00 [0.500, 45.0]	7.00 [0.500, 45.0]
Age			
18–24	2 (1.7%)	1 (0.9%)	3 (1.3%)
25–34	59 (50.4%)	54 (50.9%)	113 (50.7%)
34–44	29 (24.8%)	33 (31.1%)	62 (27.8%)
45–54	12 (10.3%)	11 (10.4%)	23 (10.3%)
55–64	9 (7.7%)	6 (5.7%)	15 (6.7%)
65–74	4 (3.4%)	1 (0.9%)	5 (2.2%)
75–84	0 (0%)	0 (0%)	0 (0%)
85 or older	0 (0%)	0 (0%)	0 (0%)
Prefer not to answer	2 (1.7%)	0 (0%)	2 (0.9%)
Ethnicity			
American Indian or Alaska Native	0 (0%)	3 (2.8%)	3 (1.3%)
Asian (Far East, Southeast Asia, Indian)	26 (22.2%)	31 (29.2%)	57 (25.6%)
Black or African American	2 (1.7%)	2 (1.9%)	4 (1.8%)
Multiple ethnicities selected	3 (2.6%)	1 (0.9%)	4 (1.8%)
Native Hawaiian or Pacific Islander	1 (0.9%)	0 (0%)	1 (0.4%)
White (Europe, Middle East, North Africa)	72 (61.5%)	56 (52.8%)	128 (57.4%)
Other	3 (2.6%)	4 (3.8%)	7 (3.1%)
Prefer not to answer	10 (8.5%)	9 (8.5%)	19 (8.5%)

*IM* internal medicine, *EM* emergency medicine, *N* numbers of participants.

### Data source and case selection

The methods used in the present study are similar to a previously published experiment^7^. The same materials from this previous study were used. The chest X-rays (frontal ± lateral projections) were sourced from the open-source MIMIC Chest X-ray database^20,21^. The Laboratory for Computational Physiology (LCP) gave explicit approval to use the X-rays in our study. A panel of three radiologists curated a set of candidate cases in an iterative process. Finally, eight cases that both reflect everyday clinical practice and test common weaknesses in chest X-ray evaluation were selected^22,23^ by a senior radiologist (EC; see online supplements for more details: https://osf.io/h7aj3/). EC added patient information, radiologic findings, proposed diagnoses, and image annotations (highlighting the area on the X-ray that leads to the diagnosis) to each case. The image IDs, patient information, radiologic findings, and diagnoses are included in the supplemental material (https://osf.io/h7aj3/). The X-rays can be found through the image IDs in the MIMIC-CXR dataset v2.0.0. To ensure that the findings, diagnoses, and image annotations, which were presented as diagnostic advice during the experiment were correct and all cases were appropriate for an assessment by physicians with different expertise levels, we pre-tested the material with six additional radiologists with varying experience levels.

### Experimental design

#### Instructions

The pre-registered (https://osf.io/sb9hf, https://osf.io/f69mz) experiment was conducted online (Qualtrics, Provo, UT). Participants were given basic information about the purpose of the study and an estimated study duration of 10 to 15 min. They were informed that participation was completely voluntary and anonymous, that they could quit the study at any time without negative consequences, and about the option of being included in a raffle as compensation for their participation. Only individuals who gave written informed consent to take part in the study (by clicking a checkbox) and confirmed that they were currently practicing radiology, internal medicine, or emergency medicine (residency included) in the USA or Canada could move on to the experiment.

#### Procedure

Next, participants learned that their task was to review and diagnose eight patient cases as accurately as possible, for which they received chest X-rays, a brief clinical history, and diagnostic advice that could be used for their final decisions. The chest X-rays were shown as a static image within the Qualtrics interface, but participants were asked to open links to an external DICOM viewer (pacsbin, Orion Medical Technologies, LLC, Towson, MD) and review the images there. The web-based DICOM viewer allowed them to adequately examine the images using all standard features of a fully functional viewing tool (e.g., zoom, window, change levels, look at annotations). We presented one example image to the participants and asked them to get familiar with the functionalities of the DICOM viewer before proceeding to review their first case. The participating physicians were asked to rate the quality of the presented diagnostic advice, give a final diagnosis, and judge how confident they were with their diagnosis. After the reviewing process was finished, participants completed a short survey, including questions about demographics, professional identification, belief in professional autonomy, self-reported AI knowledge, and attitude toward AI.

#### Manipulations

Each diagnostic advice consisted of radiologic findings and a primary diagnosis. Two characteristics of the advice were manipulated in the present experiment:

1. *Explainability of the advice*: The advice came with a visual annotation (arrows) on the chest X-rays, pointing at the area on the chest X-ray which determined the primary diagnosis. This was done to provide further explanations about the reasoning behind the advice to the participant. Arrows were chosen because existing AI-enabled clinical decision support systems (CDSS) typically use this form of annotation. By default, the annotations were automatically turned on when opening the image in the DICOM viewer. The explainability of the advice was manipulated within subjects, which means that each participant received cases with and without additional explanation. Participants received four cases with visual annotation and four without visible annotation on the X-rays. Which cases came with or without annotation was randomized.
2. *Source of the advice*: The advice was labeled as coming either from an AI-based model (CHEST-AI) or an experienced radiologist (Dr. S. Johnson). The source of the advice was manipulated between subjects; this means that participants received advice only from one source throughout the entire experiment. Receiving advice from both sources may have led the participants to adjust their quality rating based on their attitudes towards AI technology. The exact wordings for the manipulation were:
  AI: “The findings and primary diagnoses were generated by *CHEST-AI***,** a well-trained, deep-learning-based artificial intelligence (AI) model with a performance record (regarding diagnostic sensitivity and specificity) on par with experts in the field.”
  Human: “The findings and primary diagnoses were generated by *Dr. S. Johnson*, an experienced radiologist with a performance record (regarding diagnostic sensitivity and specificity) on par with experts in the field.”

Consequently, the experiment followed a 2 (*explainability of the advice*: annotated vs. not annotated) × 2 (*source of the advice*: AI vs. human) mixed factorial design to examine the effect of these two manipulations on the dependent variables (see Fig. 1).

### Measures

The present study had three dependent variables: (1) diagnostic accuracy, (2) advice quality ratings, and (3) confidence in the diagnosis:

1. *Diagnostic accuracy:* The participating physicians had to give their final diagnosis for each case by expressing whether they agreed with the diagnosis given as advice (“Do you agree with [primary diagnosis] as the primary diagnosis”). They had three response options: 1 = *full agreement* (“Yes, I agree with this diagnosis. ”), 2 = *agreement with slight modification* (“Yes, I agree with this diagnosis but would like to add a slight modification”), or 3 = *disagreement and providing an alternative diagnosis* (“No, I don't agree with this diagnosis” followed by “Please provide an alternative primary diagnosis”). Since the advice was always correct, the diagnosis was counted as 1 = accurate when the participants agreed with the advice (full agreement or agreement with slight modification). The diagnosis was counted as 0 = inaccurate when the participant disagreed with the advice.
2. *Advice quality rating:* Participants were asked to rate several aspects of the quality of the advice they received for each case. These aspects included (a) agreement ("How much do you agree with the findings?"), (b) usefulness ("How useful are the findings to you for making a diagnosis?"), (c) trustworthiness ("How much do you trust [source of advice]?"), and future consultation ("Would you consult [source of advice] in the future?"). Every question was rated on a 7-point Likert scale from 1 (*not at all*) to 7 (*extremely/definitely).* The mean of the participant’s responses to these questions was calculated to express the overall advice quality rating. The scale showed a very good internal consistency (Cronbach's α ≥ 0.87), as was already demonstrated in a previous study^7^.
3. *Confidence in the diagnosis:* For each case, participants rated the confidence in their final diagnosis with one item ("How confident are you with your primary diagnosis?") on a 7-point Likert scale from 1 (*not at all*) to 7 (*extremely*).

Several additional variables, which were considered to be relevant for this task, were measured: (**A**) *Professional identification:* Five items (e.g., "In general, when someone praises doctors, it feels like a personal compliment."^24^) were answered on a Likert scale from 1 (*strongly disagree*) to 7 (*strongly agree*); Cronbach's α = 0.74. (**B**) *Belief in professional autonomy:* Four items (e.g., "Individual physicians should make their own decisions regarding what is to be done in their work."^25^) were answered on a Likert scale from 1 (*strongly disagree*) to 7 (*strongly agree*); Cronbach's α = 0.65. (**C**) *Self-reported AI knowledge*: One item ("How would you consider your own general knowledge of artificial intelligence (AI)?”^7^) was answered on a scale from 1 (*I have no knowledge*) to 5 (*Expert knowledge*). (**D**) *Attitude toward AI:* Three items ("How much do you agree with the following statements? AI will make most people's lives better; AI is dangerous to society; AI poses a threat to my career.”^7^) were answered on a Likert scale from 1 (*strongly disagree*) to 7 (*strongly agree*); Cronbach's α = 0.61.

### Group differences

The randomization of participants into one of the two sources of the advice conditions (*N*_AI_ = 116 vs. *N*_human_ = 106) worked well. Participants in both conditions showed comparable levels of professional identification, belief in professional autonomy, self-reported AI knowledge, attitude toward AI technology, and years of professional experience (see Table S1). When divided by task expertise, the only difference among the medical fields was that radiologists rated their self-reported AI knowledge to be higher than IM/EM physicians (see Table S2). Since the experimental randomization worked and the two professional groups had similar values on all but one control variable, we ran the regressions with all participants.

## Supplementary Information

Supplementary Information.

## Data Availability

The datasets analyzed during the current study are available in the OSF repository, https://osf.io/h7aj3/.
