# Supplementary material for: Non-task expert physicians benefit from correct explainable AI advice when reviewing X-rays
Source: Sci Rep. 2023 Jan 25;13:1383. doi: 10.1038/s41598-023-28633-w (PMC9876883; doi:10.1038/s41598-023-28633-w)
Supplement: Supplementary file 1 — Supplementary Information. [file 41598_2023_28633_MOESM1_ESM.pdf]

Supplementary Materials for

Non-task expert physicians benefit from correct explainable AI advice when reviewing X-rays

Patient Case Information

Case ID: **PT001**	Pacsbin*: not annotated	MIMIC ID: p10883572\s01
• **Patient information:** A 26-year-old female presenting to the Emergency Department with chest pain.<br>• **Findings:** Normal heart size, no airspace opacification, no pleural effusion, no pneumothorax<br>• **Diagnosis:** Normal<br><br>**Description:** Case PT001 is a normal chest x-ray. The potential pitfall is misinterpreting the left breast shadow as pneumonia.<br><br>*Note: This case was removed from the statistical analysis.*		

Case ID: **PT002**	Pacsbin*: annotated, not annotated	MIMIC ID: p10165555\s04
• **Patient information:** A 51-year-old male presenting to his Primary Care Physician with chronic chest pain.<br>• **Findings:** Normal heart size, no airspace opacification, no pleural effusion, no pneumothorax, dislocated right sternoclavicular joint<br>• **Diagnosis:** Right sterno-clavicular dislocation<br><br>**Description:** Case PT002 is an uncommon fracture-dislocation injury. A systematic search pattern in interpreting chest x-rays should include the sterno-clavicular joint. Less experienced physicians may be unaware of this injury and misinterpret the fracture fragment as a pleural plaque which is a more commonly encountered finding.		

Case ID: **PT005**	Pacsbin*: annotated, not annotated	MIMIC ID: p10454165\s01
• **Patient information:** A 32-year-old female with chronic cough.<br>• **Findings:** Normal heart size, retrocardiac opacity, no pleural effusion, no pneumothorax, fourth right rib osteotomy<br>• **Diagnosis:** Hiatus hernia<br><br>**Description:** Case PT005 illustrates the importance of adjusting window levels when assessing the retrocardiac space. Normally the retrocardiac region contains traversing pulmonary vessels that taper peripherally. Careful scrutiny of this case reveals an air and soft tissue density mass in the retrocardiac region. This finding is characteristic of a hiatal hernia, a benign but potentially symptomatic entity. The cardiac silhouette on frontal chest radiographs can decrease conspicuity of important pathology that can occur in the retrocardiac space such as lung cancers, enlarged lymph nodes, and aortic aneurysms.		

*In case the DICOM cannot be viewed in Pacsbin, there are PNG versions of the original DICOM on OSF: https://osf.io/h7aj3/ *(Files -> Who should do as AI say? -> OSF Storage -> Cases)*

Case ID: **PT007**	Pacsbin*: annotated, not annotated	MIMIC ID: p10147617/s04

- **Patient information:** A 57-year-old male with shortness of breath.
- **Findings:** Mild cardiomegaly, visceral pleural edge at right apex, right basilar atelectasis, small right pleural effusion, right rib fractures
- **Diagnosis:** Right pneumothorax

Description: In case PT007, attentive interrogation of the right lung apex should have led individuals to recognize a visceral pleural edge with no distal lung markers, which are characteristic findings of a pneumothorax. It has been well established that pathology at the lung apices may be missed due to the many overlapping anatomical structures in this region.

Case ID: **PT010**	Pacsbin*: annotated, not annotated	MIMIC ID: p10165672\s10

- **Patient information:** A 63-year-old male presenting to the Emergency Department with cough.
- **Findings:** Normal heart size, right upper lobe airspace opacification, small pleural effusion, no pneumothorax
- **Diagnosis:** Right upper lobe pneumonia

Description: Case PT010 requires respondents to recognize an ill-defined right upper lung opacity. This radiographic finding and clinical history of cough should lead to the correct diagnosis of pneumonia. Respondents may have misinterpreted the ill-defined opacity as vascular markers or the superimposition of anatomical structures such as ribs.

Case ID: **PT011**	Pacsbin*: annotated, not annotated	MIMIC ID: p10176118\s01

- **Patient information:** A 46-year-old male with a 10-pack year history of smoking.
- **Findings:** Normal heart size, focal opacity projecting over right upper lung, no pleural effusion, no pneumothorax
- **Diagnosis:** Rib fracture

Description: In case PT011, a focal area of increased density appears to project over the right upper lung. A first instinct may be to consider this a pulmonary nodule. However, upon closer review, the area of increased density can be accounted for by overlapping of the third anterior and sixth posterior ribs. The correct diagnosis of an acute rib fracture can be made by identifying the step deformity of the third anterior right rib. The superimposition of anatomical structures is a well-documented cause of "pseudo-nodules".

*In case the DICOM cannot be viewed in Pacsbin, there are PNG versions of the original DICOM on OSF: https://osf.io/h7aj3/ *(Files -> Who should do as AI say? -> OSF Storage -> Cases)*

Case ID: **PT014**	Pacsbin*: not annotated	MIMIC ID: p10152675\s05
• **Patient information:** A 64-year-old male with shortness of breath.<br>• **Findings:** Moderate cardiomegaly, mild vascular redistribution, interstitial thickening, peribronchial cuffing, small bilateral pleural effusions, basilar atelectasis<br>• **Diagnosis:** Pulmonary edema<br><br>**Description:** Case PT014 requires respondents to integrate the clinical history and multiple radiograph findings to arrive at the correct diagnosis of pulmonary edema.<br>*Note: This case was removed from the statistical analysis.*		

Case ID: **PT015**	Pacsbin*: annotated, not annotated	MIMIC ID: p10426650\s01
• **Patient information:** A 19-year-old male presenting to the Emergency Department with chest pain.<br>• **Findings:** No cardiomegaly, visceral pleural edge at left apex, small left pleural effusion, left basilar atelectasis<br>• **Diagnosis:** Left pneumothorax<br><br>**Description:** In Case PT015, a less vigilant or experienced respondent may have misinterpreted the visceral pleural edge as a rib. It has been well established that pathology at the lung apices may be missed due to the many overlapping anatomical structures in this region. Attentive interrogation of the left lung apex should have led individuals to recognize a visceral pleural edge with no distal lung markers, which are characteristic findings of a pneumothorax.		

*In case the DICOM cannot be viewed in Pacsbin, there are PNG versions of the original DICOM on OSF: https://osf.io/h7aj3/ *(Files -> Who should do as AI say? -> OSF Storage -> Cases)*

Pre-Registered Study Protocols

The pre-registered study protocols (https://osf.io/sb9hf, https://osf.io/f69mz) can be found on the OSF-project page from a previously published study (https://osf.io/rjfqx/). We report two deviations: First, we planned to recruit 128 IM/EM physicians and 128 radiologists, but we ultimately only obtained 117 and 106 participants. Unfortunately, recruiting a large sample of practicing physicians for an online experiment is challenging. Second, we did not include participants' age and gender in the regression models, since we did not have a hypothesis why these demographic variables should affect the dependent variables.

Regression Model Equations

Below are the equations for the three mixed-effect regressions used for analyzing the dependent variables (1) diagnostic accuracy, (2) advice quality rating, and (3) physicians' confidence in their final diagnosis:

$$\begin{aligned}
&\mathbf{Accuracy}_{\boldsymbol{i}} \sim \text{Binomial}\,(n = 1, \text{prob}_{\text{Accuracy}=1} = \hat{P})\\
&\log\left[\frac{\hat{P}}{1-\hat{P}}\right] = \alpha_{j[i],k[i]}\\
&\qquad \alpha_j \sim N(\gamma_0^\alpha + \gamma_1^\alpha(Explainability) + \gamma_2^\alpha(Source) + \gamma_3^\alpha(Task\ expertise) +\\
&\qquad\qquad \gamma_4^\alpha(Identification) + \gamma_5^\alpha(Autonomy) + \gamma_6^\alpha(AI\ Knowledge) +\\
&\qquad\qquad \gamma_7^\alpha(AI\ Attitude) + \gamma_8^\alpha(Experience) + \gamma_9^\alpha(Explainability \times\\
&\qquad\qquad Source), \sigma_{\alpha_j}^2), \text{ for ID j} = 1, \dots, \text{J}\\
&\qquad \alpha_k \sim N(\mu_{\alpha_k}, \sigma_{\alpha_k}^2), \text{ for CASE k} = 1, \dots, \text{K}
\end{aligned} \tag{1}$$

$$\begin{aligned}
&\mathbf{Quality}_i \sim N(\alpha_(j[i], k[i]), \sigma\text{^}2)\\
&\qquad \alpha_j \sim N(\gamma_0^\alpha + \gamma_1^\alpha(Explainability) + \gamma_2^\alpha(Source) + \gamma_3^\alpha(Task\ expertise) +\\
&\qquad\qquad \gamma_4^\alpha(Identification) + \gamma_5^\alpha(Autonomy) + \gamma_6^\alpha(AI\ Knowledge) +\\
&\qquad\qquad \gamma_7^\alpha(AI\ Attitude) + \gamma_8^\alpha(Experience) + \gamma_9^\alpha(Explainability \times\\
&\qquad\qquad Source), \sigma_{\alpha_j}^2), \text{ for ID j} = 1, \dots, \text{J}\\
&\qquad \alpha_k \sim N(\mu_{\alpha_k}, \sigma_{\alpha_k}^2), \text{ for CASE k} = 1, \dots, \text{K}
\end{aligned} \tag{2}$$

$$\begin{aligned}
&\mathbf{Confidence}_{\boldsymbol{i}} \sim N(\alpha_(j[i], k[i]), \sigma\text{^}2)\\
&\qquad \alpha_j \sim N(\gamma_0^\alpha + \gamma_1^\alpha(Explainability) + \gamma_2^\alpha(Source) + \gamma_3^\alpha(Task\ expertise) +\\
&\qquad\qquad \gamma_4^\alpha(Identification) + \gamma_5^\alpha(Autonomy) + \gamma_6^\alpha(AI\ Knowledge) +\\
&\qquad\qquad \gamma_7^\alpha(AI\ Attitude) + \gamma_8^\alpha(Experience) + \gamma_9^\alpha(Explainability \times\\
&\qquad\qquad Source), \sigma_{\alpha_j}^2), \text{ for ID j} = 1, \dots, \text{J}\\
&\qquad \alpha_k \sim N(\mu_{\alpha_k}, \sigma_{\alpha_k}^2), \text{ for CASE k} = 1, \dots, \text{K}
\end{aligned} \tag{3}$$

Additional Statistical Analyses

Group differences by source of advice: Before conducting the principal statistical analysis, we checked whether the randomization of participants into the between-subjects factor *source of advice* (AI vs. human) worked. This was done by testing if there were any significant differences between the participants in both groups of advice on the variables professional identification, belief in professional autonomy, self-reported AI-knowledge, attitude toward AI, and years of experience. As Table S1 shows, the differences between the mean values of these variables were statistically non-significant for participants in the two *source of advic*e groups.

Group differences by task expertise: Additionally, we tested whether there were differences on the same variables (professional identification, belief in professional autonomy, self-reported AI-knowledge, attitude toward AI, and years of experience) between task experts (i.e., radiologists) vs. non-task experts (i.e., IM/EM physicians). The only statistically significant difference was that radiologists rated their self-reported AI knowledge higher than IM/EM physicians (see Table S2).

Table S1.
Group differences by source of advice (AI vs. human)

	AI (*N*=117)	Human (*N*=106)	*p*-value
**Professional identification**			
Mean (SD)	4.64 (0.902)	4.61 (0.982)	0.813
Median [Min, Max]	4.60 [1.80, 7.00]	4.80 [2.00, 7.00]	
**Beliefs about professional autonomy**			
Mean (SD)	3.78 (0.956)	3.79 (0.936)	0.901
Median [Min, Max]	3.88 [1.25, 6.25]	3.75 [1.75, 6.50]	
**Attitude toward AI technology**			
Mean (SD)	4.91 (0.931)	4.97 (0.971)	0.606
Median [Min, Max]	5.00 [2.67, 7.00]	5.00 [2.67, 7.00]	
**Self-reported AI-knowledge**			
Mean (SD)	2.82 (0.784)	2.89 (0.832)	0.542
Median [Min, Max]	3.00 [1.00, 5.00]	3.00 [1.00, 5.00]	
**Professional experience (years)**			
Mean (SD)	11.1 (9.79)	9.18 (9.10)	0.132
Median [Min, Max]	8.00 [0.500, 45.0]	6.00 [0.500, 40.0]	

Note. Non-task experts = internal and emergency medicine physicians (IM/EM) vs. task experts = radiologists

Table S2.
Group differences by task expertise (non-task experts vs. task experts)

	IM/EM (*N*=117)	Radiologists (*N*=106)	*p*-value
**Professional identification**			
Mean (SD)	4.57 (0.949)	4.69 (0.929)	0.348
Median [Min, Max]	4.60 [1.80, 7.00]	4.80 [2.20, 7.00]	
**Beliefs about professional autonomy**			
Mean (SD)	3.72 (0.898)	3.85 (0.994)	0.312
Median [Min, Max]	3.75 [1.25, 6.50]	4.00 [1.75, 6.25]	
**Attitude toward AI technology**			
Mean (SD)	4.90 (0.948)	4.98 (0.953)	0.541
Median [Min, Max]	5.00 [2.67, 7.00]	5.00 [2.67, 7.00]	
**Self-reported AI-knowledge**			
Mean (SD)	2.64 (0.782)	3.08 (0.770)	**<0.001**
Median [Min, Max]	3.00 [1.00, 4.00]	3.00 [1.00, 5.00]	
**Professional experience (years)**			
Mean (SD)	10.8 (9.95)	9.50 (8.96)	0.300
Median [Min, Max]	7.00 [0.500, 40.0]	6.00 [0.500, 45.0]	

Note. Non-task experts = internal and emergency medicine physicians (IM/EM) vs. task experts = radiologists

Comparing the Present Study with the Previous Study

Table S3.
Group differences: Study 1 vs. Study 2 (present study)

	Study 1 (*N*=265)	Study 2 (*N*=223)	*p*-value
**Professional identification**			
Mean (SD)	4.65 (1.02)	4.63 (0.939)	0.763
Median [Min, Max]	4.80 [1.00, 7.00]	4.70 [1.80, 7.00]	
**Beliefs about professional autonomy**			
Mean (SD)	3.89 (0.965)	3.78 (0.944)	0.223
Median [Min, Max]	4.00 [1.00, 6.50]	3.75 [1.25, 6.50]	
**Attitude toward AI technology**			
Mean (SD)	4.83 (1.01)	4.94 (0.949)	0.208
Median [Min, Max]	4.67 [1.67, 7.00]	5.00 [2.67, 7.00]	
**Self-reported AI-knowledge**			
Mean (SD)	2.92 (0.704)	2.85 (0.806)	0.351
Median [Min, Max]	3.00 [1.00, 5.00]	3.00 [1.00, 5.00]	
**Professional experience (years)**			
Mean (SD)	5.96 (7.47)	10.2 (9.49)	**<0.001**
Median [Min, Max]	3.00 [0, 37.0]	7.00 [0.500, 45.0]	

Note. Study 1: S. Gaube, H. Suresh, M. Raue, A. Merritt, S. J. Berkowitz, E. Lermer, J. F. Coughlin, J. V. Guttag, E. Colak, M. Ghassemi, Do as AI say: Susceptibility in deployment of clinical decision-aids. *npj Digital Medicine*. **4** (2021), doi:10.1038/s41746-021-00385-9.

Table S4.
Study differences between dependent variables: Study 1 vs. Study 2 (present study)

	Study 1	Study 2	*p*-value
**AI & human advice**			
**Diagnostic accuracy**			
Mean (SD)	82.4 (38.1)	85.4 (35.3)	0.088
Median [Min, Max]	100 [0, 100]	100 [0, 100]	
**Advice quality rating**			
Mean (SD)	5.12 (1.26)	5.27 (1.24)	**0.013**
Median [Min, Max]	5.25 [1.00, 7.00]	5.50 [1.00, 7.00]	
**Confidence rating**			
Mean (SD)	5.55 (1.26)	5.66 (1.22)	0.063
Median [Min, Max]	6.00 [1.00, 7.00]	6.00 [1.00, 7.00]	
**Only AI advice**			
**Diagnostic accuracy**			
Mean (SD)	85.0 (35.7)	88.4 (32.1)	0.137
Median [Min, Max]	100 [0, 100]	100 [0, 100]	
**Advice quality rating**			
Mean (SD)	4.96 (1.21)	5.28 (1.17)	**<0.001**
Median [Min, Max]	5.00 [1.00, 7.00]	5.50 [1.00, 7.00]	
**Confidence rating**			
Mean (SD)	5.62 (1.21)	5.76 (1.21)	0.102
Median [Min, Max]	6.00 [1.00, 7.00]	6.00 [1.00, 7.00]	
**Only human advice**			
**Diagnostic accuracy**			
Mean (SD)	79.7 (40.3)	82.1 (38.4)	0.377
Median [Min, Max]	100 [0, 100]	100 [0, 100]	
**Advice quality rating**			
Mean (SD)	5.28 (1.29)	5.26 (1.32)	0.817
Median [Min, Max]	5.50 [1.00, 7.00]	5.50 [1.25, 7.00]	
**Confidence rating**			
Mean (SD)	5.47 (1.29)	5.55 (1.23)	0.356
Median [Min, Max]	6.00 [1.00, 7.00]	6.00 [1.00, 7.00]	

Note. Study 1: S. Gaube, H. Suresh, M. Raue, A. Merritt, S. J. Berkowitz, E. Lermer, J. F. Coughlin, J. V. Guttag, E. Colak, M. Ghassemi, Do as AI say: Susceptibility in deployment of clinical decision-aids. *npj Digital Medicine*. **4** (2021), doi:10.1038/s41746-021-00385-9.

Regression Results without Cases PT002 and PT015

Table S5.

Logistic mixed multilevel regression models for participants' diagnostic accuracy (without PT002 and PT015).

Predictors	*Odds Ratios*	*SE*	*95% CI*	*z*	*p*
Intercept	3.77	2.07	1.29 – 11.05	2.42	**0.015**
Explainability [annotated]	1.85	0.53	1.05 – 3.26	2.14	**0.033**
Source [AI]	1.79	0.52	1.01 – 3.17	2.00	**0.045**
Task expertise [experts: radiologists]	2.13	0.50	1.34 – 3.38	3.21	**0.001**
Professional identification	0.96	0.11	0.77 – 1.21	-0.31	0.760
Beliefs about professional autonomy	1.15	0.14	0.91 – 1.46	1.18	0.237
Self-reported AI-knowledge	1.35	0.20	1.02 – 1.80	2.07	**0.039**
Attitude toward AI	1.00	0.12	0.79 – 1.28	0.03	0.978
Professional experience (years)	0.99	0.01	0.97 – 1.02	-0.53	0.599
Explainability [annotated] x Source [AI]	0.68	0.28	0.30 – 1.51	-0.95	0.340

Note. SE = standard error; *p* = probability of committing a Type I error; random effects: σ^2 =3.29, $\tau_{00\ ID}$ = 0.40, $\tau_{00\ PATIENTID}$ = 0.94, ICC = 0.29, N_{ID} = 222, $N_{PATIENTID}$ = 4, Observations = 888, Marginal R^2 = 0.072 / Conditional R^2 = 0.340; OR>1 variable associated with higher odds for correct diagnosis; OR<1 variable associated with lower odds for correct diagnosis, OR=1 variable does not affect odds of outcome. The intercept indicates that the probability of an accurate diagnosis was 0.79 when all predictors are zero. Predictors without a natural zero point (i.e., professional identification, beliefs about professional autonomy, self-reported AI-knowledge, attitude toward AI) were mean-centered.

Table S6

Linear mixed multilevel regression models for advice quality rating (without PT002 and PT015).

Predictors	*Estimate*	*SE*	*95% CI*	*t*	*p*
Intercept	4.99	0.25	4.50 – 5.48	19.98	**<0.001**
Explainability [annotated]	0.20	0.10	0.01 – 0.39	2.09	**0.037**
Source [AI]	-0.00	0.13	-0.25 – 0.24	-0.01	0.993
Task expertise [experts: radiologists]	0.33	0.11	0.12 – 0.55	3.04	**0.002**
Professional identification	0.03	0.06	-0.08 – 0.14	0.49	0.626
Beliefs about professional autonomy	-0.11	0.06	-0.23 – 0.00	-1.96	0.050
Self-reported AI-knowledge	0.08	0.07	-0.05 – 0.22	1.22	0.222
Attitude toward AI	0.13	0.06	0.02 – 0.25	2.33	**0.020**
Professional experience (years)	-0.00	0.01	-0.01 – 0.01	-0.11	0.910
Explainability [annotated] x Source [AI]	-0.02	0.13	-0.28 – 0.24	-0.18	0.858

Note. SE = standard error; *p* = probability of committing a Type I error; random effects: σ^2 =0.89, $\tau_{00\ ID}$ = 0.39, $\tau_{00\ PATIENTID}$ = 0.19, ICC = 0.40, N_{ID} = 222, $N_{PATIENTID}$ = 4, Observations = 888, Marginal R^2 = 0.051 / Conditional R^2 = 0.427. The regression estimate indicates how much the mean quality rating changes given a one-unit shift in the predictor while holding other predictors in the model constant. The intercept represents the mean value of the advice quality rating when all predictor variables are zero. Predictors without a natural zero point (i.e., professional identification, beliefs about professional autonomy, self-reported AI-knowledge, attitude toward AI) were mean-centered.

Table S7.
Linear mixed multilevel regression models for confidence in the diagnosis (without PT002 and PT015).

Predictors	*Estimate*	*SE*	*95% CI*	*t*	*p*
Intercept	4.96	0.25	4.47 – 5.44	20.10	**<0.001**
Explainability [annotated]	-0.04	0.10	-0.23 – 0.15	-0.40	0.690
Source [AI]	0.14	0.11	-0.08 – 0.37	1.25	0.211
Task expertise [experts: radiologists]	0.74	0.10	0.55 – 0.93	7.76	**<0.001**
Professional identification	0.01	0.05	-0.09 – 0.10	0.14	0.888
Beliefs about professional autonomy	0.02	0.05	-0.08 – 0.12	0.37	0.713
Self-reported AI-knowledge	0.21	0.06	0.09 – 0.33	3.43	**0.001**
Attitude toward AI	0.00	0.05	-0.10 – 0.10	0.05	0.959
Professional experience (years)	0.01	0.00	0.01 – 0.02	3.01	**0.003**
Explainability [annotated] x Source [AI]	0.04	0.14	-0.22 – 0.31	0.31	0.753

Note. SE = standard error; *p* = probability of committing a Type I error; random effects: σ^2 =0.93, $\tau_{00\ ID}$ = 0.23, $\tau_{00\ PATIENTID}$ = 0.20, ICC = 0.31, N_{ID} = 222, $N_{PATIENTID}$ = 4, Observations = 888, Marginal R^2 = 0.140 / Conditional R^2 = 0.409. The regression estimate indicates how much the mean confidence rating changes given a one-unit shift in the predictor while holding other predictors in the model constant. The intercept represents the mean value of the confidence in the diagnosis when all predictor variables are zero. Predictors without a natural zero point (i.e., professional identification, beliefs about professional autonomy, self-reported AI-knowledge, attitude toward AI) were mean-centered.
